# A decade of child dental health in the Falkland Islands, 2013–2025

**DOI:** 10.1038/s41415-026-9618-6

**Published:** 2026-06-12

**Authors:** Colwyn M. Jones, James W. Edwards

**Affiliations:** 699596234290350264851 School of Dentistry, Park Place, University of Dundee, United Kingdom; 828033745300147081327 Dental Department, King Edward VII Memorial Hospital, Stanley, Falkland Islands

## Abstract

**Introduction** The Falkland Islands are a remote archipelago of over 740 islands and host a diverse, multicultural society. Since 2013, consistent efforts to improve and protect children's oral health have been applied and evaluated, and the results after a decade are presented.

**Methods** Child dental health surveys aligning with the World Health Organization methodology have been conducted annually since 2013 (exceptions: 2015 and 2020). Data analysis was undertaken to establish a mean dmft/DMFT (decayed, missing, and filled primary teeth/decayed, missing, and filled permanent teeth) for five-year-olds, 12-year-olds and 15-year-olds within the Falkland Islands.

**Results** Mean dmft/DMFT results have trended down since 2013, with all age groups demonstrating a dmft/DMFT of below 1.0 tooth consistently since 2021. Response rates have been very good to excellent (69% to 97%) which increases confidence in the results.

**Conclusions** Preventative interventions within the Falkland Islands appear to be improving child dental health. There is further scope, and need, for improvement, and additional screening should be investigated to understand the current oral health of the whole population of the Falkland Islands.

## Key points

- We report ten-year trends in child dental health surveys of the Falkland Islands which meets World Health Organization targets.
- Tooth decay experience of children in the Falkland Islands has gradually improved over the study period.
- Child dental health of the Falkland Islands is good and comparable with the best Northern European countries.

## Introduction

The Falkland Islands are one of 14 UK Overseas Territories and form an archipelago in the South Atlantic Ocean off the eastern coast of South America, approximately 8,000 miles south-east of the UK. The Islands are perhaps best known for the Falklands War, a 74-day conflict between Argentina and the UK, which ended on 14 June 1982.^1^ A referendum was held in 2013, in which the local population reaffirmed their British sovereignty.^2^^,^^3^

The archipelago of over 740 islands has a land area of approximately 4,700 square miles (12,173 km²);^4^ roughly half the size of Wales. The capital city, Stanley, located on East Falkland, is home to approximately 75–80% of the Islands' population (n = 2,538 *cf.* n = 3,541 in 2021).^5^

The remote location and variable climate make provision of fresh food, particularly fruits and vegetables, unpredictable. The Falkland Islands Government (FIG) run a market garden/farm shop^6^ which is a major source of fresh produce, both locally grown and imported. Meat and eggs are typically easier to come by: the areas outside of Stanley, referred to as ‘Camp', are essentially remote countryside. Camp hosts a number of local farms which supply the population of the Falklands with beef, mutton, and eggs.

The economy of the Falkland Islands originally involved sealing, whaling and provisioning ships rounding Cape Horn, and later became heavily dependent on sheep farming from the 1870s to 1980. Since then, it has become more diverse with more income from tourism and commercial fishing but retains livestock (mainly sheep) farming.

Dental (and medical) services are based in the King Edward VII Memorial Hospital (KEMH) in Stanley. The hospital hosts a team of doctors who act as general practitioners, ward doctors and the on-call medical team, along with the support of the nurses. They care for the entire population of the Islands, from routine management of chronic conditions up to emergency presentations at the casualty unit. Healthcare providers at KEMH are either international contractors (on fixed-term contracts of up to four years' duration) or local residents, typically on permanent contracts. There is a wide range within this, with some specialties rotating as often as every twelve weeks due to full-time on-call commitments.

Working in a small, remote hospital with limited resources does restrict the type of care that can be offered, and patients may need to be referred on for diagnostics or treatment that is beyond the scope of a small remote health service. In these cases, patients are typically referred to UK hospitals under a reciprocal agreement with the National Health Service (NHS). In life-threatening situations medical evacuation of patients to larger hospitals in nearby South America is undertaken. Visiting specialists can improve access to care locally, including visiting specialist urological, gastroenterological and maxillofacial surgeons, amongst others.

The dental department is staffed by up to three clinicians, four nurses, and a ‘department facilitator'– a job role tailored to the needs of the department which encompasses some duties of a practice manager, lead dental nurse, and administrator. While located within the KEMH building, the department runs like a conventional UK general dental practice with routine recalls and examinations, planned dental care, and emergency slots each day. The scope of each clinician working in the department is, by necessity, wider than it would be within the UK. With the easiest accessible dental technicians being via air cargo to the UK, a small laboratory facility at the KEMH allows clinicians to cast models from impressions, and make special trays, occlusal registration rims, retainers, night-guards, as well as repair and add teeth to dentures. The responsibility for Community Dental Services also falls to the department, who carry out semi-annual screenings at the Islands' local prison, care home, and infant and senior schools. More recently, a local orthodontic service^7^ was established relying on a tele-dentistry link between the KEMH dental team and a remote UK-based specialist responsible for screening and treatment planning. Emergency dental care is available year-round, including a non-working day on-call rota. While this is predominantly accessed by locals (or longer-term visitors, i.e., those on longer work contracts), the service also extends to visiting tourists and crew on shipping or fishing vessels.

In 2015, a local initiative aligned with the principles of the Childsmile programme^8^ was established, with daily ‘brush-ins' at the nursery. For their first birthday, every child should receive a toothbrush, and all schoolchildren are invited to attend a semi-annual fluoride varnish clinic run at the school by an additionally qualified dental nurse or a dental officer.

The earliest epidemiological data on oral health in the Falkland Islands are from the year 2000, revealing a national dmft (decayed, missing, and filled primary teeth) of approximately five teeth per five-year-old as being decayed, missing, or filled, and remarks on the ‘terrible' rate of full dental clearances.^9^ The dmft value was estimated from available clinical records: an attempt at an innovative approach, but the results were an estimate with unclear accuracy and which we cannot confidently generalise to the wider population at the time.

Until 2013, there were scant data on child dental health in the Falkland Islands. This was addressed by a school screening and child dental health survey programme, developed to align with the guidance from the World Health Organization (WHO).^10^ Every schoolchild is offered a place on the school screening visits which take place approximately once every 12 months. Child dental health survey screenings sample the five-, 12- and 15-year-old children, in line with WHO guidelines, and follow the published methodological guidelines.^10^ It is these child dental health surveys which generated the data for this report.

Results from the first dental survey (2013) were reported in 2015, demonstrating levels of child dental decay experience in the Falkland Islands which were ‘similar to Western European countries'.^11^ Now with a decade of data to draw upon, we can present trends in the oral epidemiological survey results from the Falkland Islands.

## Methods

Each year, lists of enrolled pupils were provided to the dental department by the education department of FIG. From this, children eligible for the child oral health survey were identified and invited to participate. Written consent was obtained from the parents/guardians of children who participated, with positive assent also obtained on the day from each participant.

Screening was carried out in a separate room of the school under ambient lighting, using just a mirror and cotton rolls to dislodge food or debris, but only visual (not tactile) inspection was undertaken

The transient nature of staffing in the dental department at KEMH means staff have variable experience of running epidemiological surveys; however, in-house training is provided to each staff member undertaking the data collection. Currently, there is no formalised calibration of examiners excepting those who have previously calibrated in the UK.

Data were collected contemporaneously on paper forms, then collated into a spreadsheet to calculate the mean age, response rate, mean dmft/DMFT (decayed, missing, and filled primary/permanent teeth) and proportion free of caries experience (%dmft/DMFT = 0) for each of the three age groups.

No research ethics committee exists in the Falkland Islands, therefore ethical approval to publish these results was granted by the Directorate of Health and Social Services of FIG.

Data analysis and presentation was conducted with Microsoft Excel (Microsoft Excel, Version 2506, 2025).

## Results

Dental epidemiological surveys were carried out annually between 2013 and 2025, with the exception of 2015 (due to critical staffing shortages) and 2020 (during the SARS-CoV-2 pandemic).

The number of children surveyed across the year and age groups varied from 21–54, reflecting the annual birth cohort of Falkland Islands residents, plus any resident children of longer-term contractors. High response rates (69–97%) approach a census survey.

### Dental health of five-year-old children

The mean dmft of five-year-old children on the Falklands has been consistently below 1.0 tooth since 2019 (see Table 1).

**Table 1:** Dental epidemiological survey results of five-year-old children, 2013–2025

**Year**	**2013**	**2014**	**2015**	**2016**	**2017**	**2018**	**2019**	**2020**	**2021**	**2022**	**2023**	**2024**	**2025**
Examined (n)	26	36	-	31	29	29	35	-	33	33	23	22	30
Response rate (%)	79	88	-	93	94	83	95	-	83	85	92	71	91
Mean age (yrs)	5.5	5.48	-	5.42	5.44	5.49	5.52	-	5.6	5.47	5.62	5.47	5.59
Mean dmft	1.2	0.53	-	1.03	1.13	1.07	0.66	-	0.97	0.88	0.78	0.83	0.8
dmft = 0 (n)	17	28	-	24	19	16	30	-	23	24	19	19	22
dmft = 0 (%)	65	78	-	77	66	55	86	-	70	73	83	86	73
dmft, decayed, missing and filled primary teeth													

### Dental health of 12-year-old children

No data existed for 12-year-old children on the Falkland Islands before 2013.

The mean DMFT of 12-year-old children on the Falklands has been consistently below 1.0 tooth since the first survey in 2013 (see Table 2).

**Table 2:** Dental epidemiological survey results of 12-year-old children, 2013–2025

**Year**	**2013**	**2014**	**2015**	**2016**	**2017**	**2018**	**2019**	**2020**	**2021**	**2022**	**2023**	**2024**	**2025**
Examined (n)	31	26	-	42	26	38	25	-	40	54	34	32	50
Response rate (%)	97	96	-	96	79	95	89	-	95	95	92	94	96
Mean age (yrs)	12.5	12.4	-	12.5	12.4	12.4	12.5	-	12.5	12.5	12.56	12.5	12.54
Mean DMFT	0.9	0.76	-	0.43	0.3	0.63	0.79	-	0.25	0.38	0.50	0.59	0.50
DMFT=0 (n)	19	17	-	31	21	27	16	-	33	46	27	23	39
DMFT = 0 (%)	63	65	-	74	81	71	64	-	83	85	79	72	78
DMFT, decayed, missing and filled permanent teeth													

### Dental health of 15-year-old children

As with the 12-year-old group, no data exist prior to 2013 for the dental health of 15-year old children.

The dental health of the 15-year-olds has fallen (improved) from a mean of close to 2.0 in 2013, to consistently below 1.0 tooth from 2021 to 2025 (see Table 3).

**Table 3:** Dental epidemiological survey results of 15-year-old children, 2013–2025

**Year**	**2013**	**2014**	**2015**	**2016**	**2017**	**2018**	**2019**	**2020**	**2021**	**2022**	**2023**	**2024**	**2025**
Examined (n)	27	21	-	27	23	29	39	-	25	39	25	26	41
Response rate (%)	87	90	-	93	88	83	89	-	83	93	76	69	89
Mean age (years)	15.4	15.5	-	15.6	15.5	15.4	15.4	-	15.4	15.5	15.5	15.3	15.59
Mean DMFT	1.78	0.88	-	0.93	1.04	1.15	1.16	-	0.52	0.49	0.36	0.85	0.95
DMFT = 0 (n)	9	13	-	18	13	15	23	-	19	28	20	16	26
DMFT = 0 (%)	33	62	-	67	57	52	79	-	76	72	80	62	63
DMFT, decayed, missing and filled permanent teeth													

**Fig. 1:**
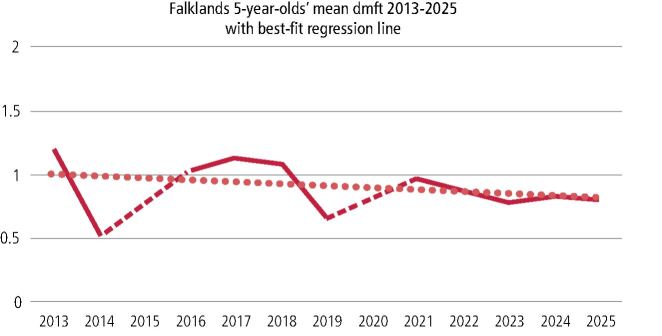
Mean dmft of five-year-old children in the Falkland Islands, 2013–2025

**Fig. 2:**
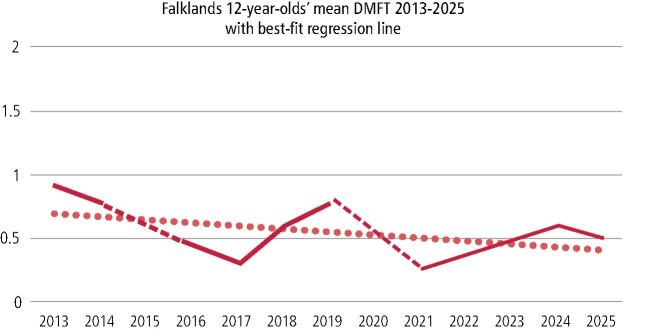
Mean dmft of 12-year-old children in the Falkland Islands, 2013–2025

**Fig. 3:**
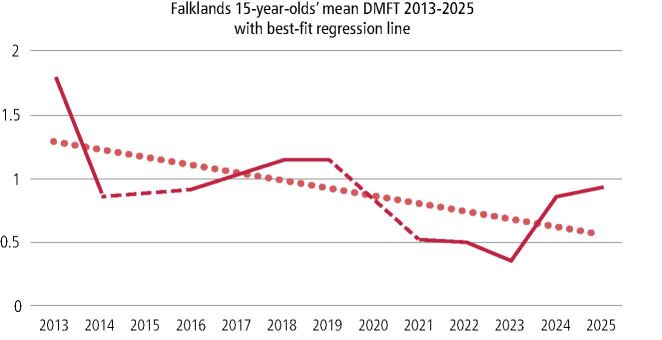
Mean DMFT of 15-year-olds in the Falkland Islands, 2013–2025

Figures (Fig. 1, Fig. 2 and Fig. 3) showing the dental health for each age group from 2013–2025 have the unweighted best fit regression lines added to indicate the trends over this period. In all three age groups the regression line equations (Table 4) have a negative gradient indicating an overall reduction in mean dmft/DMFT. These equations correspond to progressively steeper declines in mean dmft/DMFT within older age cohorts, indicating that values have improved (reduced) more within 15-year-olds than 12-year-olds, and both have improved (reduced) more than five-year-olds.

**Table 4:** Regression line equations for dental health trends in five-year-olds, 12-year-olds and 15-year-olds in the Falkland Islands between 2013–2025

**Age group**	**Regression line equation**
Five-year-olds	*y* = -0.0141x + 1.0007
12-year-olds	*y* = -0.0245x + 0.6949
15-year-olds	*y* = -0.0606x + 1.3602

## Discussion

The results from our surveys show that child dental health trends over ten years from 2013 to 2025 compare favourably with results from the dentally healthiest Northern European countries^12^ and also shows a similar improvement over this period.^12^

Since 2021, the Falklands have consistently aligned with the WHO ‘developed nations' target^13^^,^^14^^,^^15^ of a mean DMFT of less than one tooth by the year 2000. The consistency of the mean dmft/DMFT result for the three age groups over time give us confidence in the reliability of these surveys, but the small numbers of children in each year cohort perhaps account for the annual variation seen.

While the figures show an improving trend in child dental health for five-, 12- and 15-year-olds, tooth decay is a chronic disease with each new generation at risk of developing decay. The regression data suggest that, over the ten-year reporting period, 15-year-olds' DMFT has improved at approximately twice the rate of the 12-year-olds', and 12-year-olds' DMFT has improved at approximately twice the rate of five-year-olds' dmft. Despite variable annual results, the trend in average dmft/DMFT is undeniably positive. Sustained multidimensional health improvement initiatives are required to improve dental health;^16^ therefore, to prevent a resurgence of caries and perhaps maintain existing improvements in dental health, initiatives should be maintained and new approaches implemented and continued to further reduce the burden of poor oral health on the population of the Falkland Islands. There is value in evidence-based initiatives such as a local soft drinks industry levy (SDIL or ‘sugar tax'),^17^ community water fluoridation,^18^ and advertising restrictions on confectionary.^19^

The five risk factors for poor oral health (oral hygiene, fluoride, diet, tobacco, and alcohol) represent a wide opportunity for intervention to safeguard oral health. Initiatives addressing these multiple oral health risk factors are also likely to improve general health, therefore a spectrum of interventions across the risk factors is recommended.

This common risk factor approach may help to address other local public health concerns. For example, obesity is prevalent in the local population. Addressing dietary habits, especially intake of sugar and sugar-sweetened beverages, would present potential improvements in oral health and obesity.

Mid-stream and upstream interventions generate wider impacts, so alongside tailored advice on the oral health risk factors for individual patients, wider policy changes and public health interventions are better for the health of a population.

Untapped and upstream multi-agency interventions such as community water fluoridation, SDIL and healthy eating standards in schools would necessitate the involvement of the dental department, the public works department, hospital finance managers, the local political system, the education department, broader healthcare workers, and would also involve international collaboration with experts in the field.

In addition, little is known about the standard of oral health amongst the adult population. An adult oral health survey would help to develop a more complete picture of the efficacy of preventative interventions within the Falkland Islands and inform further steps to safeguard the oral health of the entire population.

## Conclusion

The oral health of five-, 12- and 15-year-olds in the Falkland Islands has improved since 2013 with the average dmft/DMFT for every age group reducing substantially. Since 2021, an average dmft/DMFT of less than 1.0 has been maintained for every age group. The biggest change has been seen in 15-year-olds with a 46% reduction in average DMFT between 2013–2025. These data suggest that preventative initiatives that are reaching children and parents in the Falkland Islands are helping to protect children's dental health, but that there is room for further improvement in national oral health.

## Data Availability

The full anonymised dataset is available from the corresponding author on request.
